# No Effect of Low-Dose Glucocorticoid Maintenance Therapy on Damage in SLE Patients in Prolonged Remission: A Propensity Score Analysis of the Longitudinal Lupus-Cruces-Bordeaux Inception Cohort

**DOI:** 10.3390/jcm13206049

**Published:** 2024-10-11

**Authors:** Guillermo Ruiz-Irastorza, Diana Paredes-Ruiz, Luis Dueña-Bartolome, Halbert Hernandez-Negrin, Victor Moreno-Torres, Christophe Richez, Estibaliz Lazaro

**Affiliations:** 1 Biobizkaia Health Research Institute, Hospital Universitario Cruces, 48903 Barakaldo, Spain; diana.paredesruiz@osakidetza.eus (D.P.-R.); luisfrancisco.duenabartolome@osakidetza.eus (L.D.-B.); halberthn@gmail.com (H.H.-N.); victor.moreno.torres.1988@gmail.com (V.M.-T.); 2 Faculty of Medicine UPV/EHU, 48940 Leioa, Spain; 3 Instituto de Investigación Biomédica de Málaga (IBIMA-Plataforma BIONAND), Hospital Regional Universitario de Málaga, 29010 Málaga, Spain; 4 Faculty of Medicine, Universidad de Málaga, 29010 Málaga, Spain; 5 UNIR Health Sciences School and Medical Center, 28224 Pozuelo de Alarcón, Spain; 6 Systemic Autoimmune Diseases Unit, Hospital Universitario Puerta de Hierro Majadahonda, 28222 Majadahonda, Spain; 7 Department of Internal Medicine, Bordeaux University Hospital, FHU ACRONIM, 33000 Bordeaux, France; christophe.richez@chu-bordeaux.fr (C.R.); estibaliz.lazaro@chu-bordeaux.fr (E.L.)

**Keywords:** glucocorticoids, damage, systemic lupus erythematosus, hydroxychloroquine, remission

## Abstract

**Background/Objectives**: Prolonged remission on low-dose glucocorticoids (GC) is a main goal in patients with systemic lupus erythematosus (SLE). The aim of this study is to assess whether GC ≤ 5 mg/d increases the risk of damage accrual in patients with SLE in prolonged remission. **Methods**: Observational study of routine clinical care data of the inception Lupus Cruces-Bordeaux cohort. Only patients in DORIS remission during five consecutive yearly visits were included. The endpoint was damage accrual during the 5-year follow-up, either global or specific damage: GC-induced, cardiovascular (CV), lupus and other. Patients no longer on GC therapy by year 5 (GC5-Off) were compared with those who continued GC therapy (GC5-On). Comparisons were made by Cox and Poisson regressions, which were adjusted with propensity score (PE) in order to control for confounding by indication. **Results**: 132 patients were included, 56 in the GC5-On and 76 in the GC5-Off groups. All patients were on GC ≤ 5 mg/d for the whole follow-up, the mean prednisone dose in the GC5-On group being 2.96 mg/d during the whole study period and 2.6 mg/d during the 5th year. Fourteen patients (10.6%) accrued damage. More patients in the GC5-On group accrued global damage, 16% vs. 7% in the GC5-Off group, *p* = 0.08, mainly at CV domains (7% vs. 1%, respectively, *p* = 0.16). In the PS-adjusted Cox and Poisson regressions, the GC5-On group was not significantly associated with global (*p* = 0.39) or CV damage accrual (*p* = 0.62), nor with the absolute (*p* = 0.40) or CV-restricted final SDI scores (*p* = 0.63). The C-index of the propensity score model was 0.79. **Conclusions**: Maintaining doses of prednisone < 5 mg/d in lupus patients in prolonged remission is not associated with an increased risk of damage accrual.

## 1. Introduction

Damage and remission are considered crucial concepts in systemic lupus erythematosus (SLE). Damage, defined as irreversible organ dysfunction, related to either the disease process or to its therapy, is a major prognostic variable [1,2]. The concept of remission has long been difficult to standardize, but recently, the DORIS (Definitions of Remission in SLE) definition, which includes a clinical SLEDAI = 0 [3], has demonstrated achievable [4] and also protective against damage accrual [5]. Therefore, both the achievement of DORIS remission and the prevention of damage are among the main targets of the 2023 EULAR guidelines [6].

Glucocorticoids (GC) are a main determinant of damage accrual [7] and, despite the recommendations of long-term doses of prednisone ≤ 5 mg/d (EULAR), the exact safety threshold has not been well established [7]. However, early discontinuation of GC (now considered a “bridging therapy”) is advised, with the recommendation of introducing additional therapies, including biologic drugs, in case this goal cannot be achieved [6].

DORIS remission includes patients with prednisone up to 5 mg/d, therefore also individuals on no GC [3]. In a recent study within the SLICC cohort in which the different definitions for remission were mutually exclusive (i.e., patients on clinical remission with prednisone >0 to ≤5 mg/d were compared with those on no GC), damage accrual was not different between both groups [5]. A recent meta-analysis of studies comparing GC withdrawal vs. low-dose maintenance suggested a small increase in damage among users but also an increase in activity among those stopping GC [8]. Therefore, we are still missing the whole picture. More specifically, there are limited data directly comparing the long-term safety of maintaining prednisone at ≤5 mg/d versus complete withdrawal in patients meeting DORIS remission criteria.

In a recent study of the Cruces-Bordeaux inception Lupus cohort, we observed high rates of DORIS remission during 5 consecutive years after the diagnosis [9], thus providing us with an optimal environment to study the effects of GC withdrawal vs. maintenance of low doses on damage accrual in the absence of lupus activity.

## 2. Materials and Methods

### 2.1. Study Design and Patients

This is an observational study of routine clinical care data of the international Lupus Cruces-Bordeaux cohort, as described in previous studies [4,9]. The longitudinal design of the Lupus Cruces-Bordeaux cohort, with rigorous yearly follow-ups, provides robust real-world evidence on the effects of long-term low-dose GC. Eligible patients fulfilled the SLICC criteria for the classification of SLE [10]. The point when classification criteria were met was considered time 0 of follow-up (T0). All inception patients diagnosed between years 2000 and 2017, treated since T0 at the respective centers with 5 years of follow-up were included [9]. Patients who died (*n* = 1) or were lost to follow-up (*n* = 3) before the completion of the 5-year follow-up were excluded from the analysis [9].

For this study, we selected from the database those patients who achieved prolonged remission according to DORIS definition, i.e., remission criteria were fulfilled in each of the 5 yearly visits after T0 [3,9]. Therefore, the influence on damage accrual of SLE activity and of mid-long-term exposure to medium-high doses of GC was minimized.

Data were updated upon the cohort entry and then once a year, counting after T0, with additional information retrieved from the electronic records of each patient, where structured clinical and therapeutic information is contained. For therapeutic variables, we recorded its use (or not) and the cumulative dosage within each year of follow-up, which means that the exact point of administration of individual treatments was not available.

The protocol of the prospective observational lupus cohort was approved by Biobizkaia research board (Code BC/A/23/010, 31 March 2009) in compliance with the Declaration of Helsinki. All participants signed an informed consent form on joining the cohort.

### 2.2. Outcome Measures

The main endpoint of this study is damage accrual during the 5-year follow-up, according to the SDI [1]. The variables used for the analysis of general damage were whether any damage was present (categorized as yes/no) and the SDI absolute score at the last follow-up visit. In order to analyze the specific types of damage attributable to different causes, the SDI subcategories were grouped as follows [1,11]: (1) SLE-related damage, including any of the following SDI items: cognitive impairment or psychosis, myelitis, neuropathy (excluding optic atrophy), renal damage (proteinuria ≤ 3.5 g/24 h, estimated or measured GRF < 50% or end stage renal disease, regardless of dialysis or transplantation), pulmonary or pleural fibrosis, shrinking lung, chronic pericarditis, tissue loss, chronic peritonitis, deforming or erosive arthritis, ruptured tendons, alopecia, extensive scarring or skin ulceration; (2) GC-related damage: cataracts, muscle atrophy or weakness, osteoporosis with fracture or vertebral collapse, avascular necrosis or diabetes; (3) CV damage: cerebrovascular accident, angina or coronary bypass, myocardial infarction, ventricular dysfunction or claudication; (4) Unclassified damage, a heterogeneous group formed by SDI items not included in the previous groups: retinal change or optic atrophy, seizures, pulmonary hypertension, pulmonary infarction or resection, valvular disease, venous thrombosis with swelling, ulceration or stasis, bowel-spleen-liver or gall bladder resection, mesenteric or pancreatic insufficiency, upper intestinal surgery, osteomyelitis, premature gonadal failure and malignancy.

### 2.3. Independent Variables

We explored the baseline clinical profile potentially impacting on damage accrual by using the following variables: center (Cruces or Bordeaux); gender; White/other race; age at diagnosis; presence of cardiovascular risk factors (smoking, hypertension, dislipemia and diabetes mellitus); and entry SLEDAI-2K [12], both as the absolute score and as categories of activity: mild (SLEDAI < 6), moderate (SLEDAI 6-12) and severe (SLEDAI > 12) activity [6]. We also recorded the following therapeutic variables within the first 12 months after T0: average prednisone daily dose (mg/d), use of methyl-prednisolone pulses (MP), cyclophosphamide (CYC); oral immunosuppressive drugs (azathioprine, methotrexate, mycophenolate, calcineurin inhibitors); and biologic drugs (belimumab or rituximab). In addition, we included the cumulative number of months on HCQ during the 5-year follow-up as a quantitative measure of the exposure to HCQ, given its long-term effects on preventing damage.

### 2.4. Statistical Analysis

We identified those patients who were no longer on GC therapy by the end of year 4 (group named GC5-Off), who were compared for the outcomes with those who continued with GC therapy during year 5 (group named GC5-On).

Descriptive data were generated, using percentages, means and standard deviations (SD). Baseline variables as well as GC exposure were compared between GC5-On and GC5-Off groups. Data were compared by Chi-squared test, two-tailed Fischer exact test or student *t*-test, as appropriate.

The potential influence of GC withdrawal by year 5 on damage was analyzed by several means. The proportions of patients in the GC5-On and GC5-Off accruing any damage or specific damage in each of the SDI subcategories (i.e., SLE-related, GC-related, CV or other damage) were compared. Given the skewed distribution of the final global and CV-restricted SDI scores, a Poisson regressions was used to analyze the association between both and the GC5 groups.

For adjustment, a propensity score (PS) analysis was performed in order to minimize the possibility of bias caused by confounding by indication [13]. The individual PS, that is, the probability of having GC continued by year 5, was calculated for each patient by using a logistic regression model, in which center, gender, White vs. non-White race, age at diagnosis, the presence of CV risk factors (smoking, hypertension, dyslipidemia and/or diabetes), aPL positivity, baseline SLEDAI 2K score and therapeutic variables during the first year (average prednisone dose, use of MP and oral immunosuppressive drugs) were the independent predictors. CYC was excluded from the analysis because it predicted failure (i.e., damage accrual) perfectly. Likewise, time on HCQ was not included given the almost total follow-up time on this treatment in both groups (see Section 3). Biologics were not incorporated into the PE calculation since no patients in this group received rituximab or belimumab within the first year of follow-up. The discriminatory capacity of this model was assessed by the area under the receiver operating characteristic (ROC) curve (C-index).

The resulting PS was then included as an adjustment covariate in a Cox proportional hazard model with years to first global and CV damage accrual as the time variable. To complete the analysis, PS adjusting was added to both Poisson regressions using the global and CV-restricted SDI scores.

The statistical analysis was performed using STATA/MP 14.2 for Mac (StataCorp LP, College Station, TX, USA).

## 3. Results

### 3.1. Study Group

Out of 233 patients included in the joined inception Lupus-Cruces-Bordeaux database, 132 (57%) achieved prolonged remission (8). As expected, damage accrual was more frequent among patients not achieving prolonged remission (26.7% vs. 10.6%, *p* = 0.001).

Among the 132 patients, 43 (33%) had stopped prednisone by the end of year 1, 54 patients (41%) by the end of year 2, 63 patients (48%) by the end of year 3 and 76 (58%) by the end of year 4, the remaining 56 patients entering year 5 on GC therapy. Therefore, the GC5-On and GC5-Off groups were finally formed by 56 and 76 patients, respectively. The main characteristics of both groups are shown in Table 1. Patients in the GC5-On group presented more severe disease (mean baseline SLEDAI 6.3 vs. 4.6, respectively, *p* = 0.01) and, accordingly, were treated with higher doses of prednisone, more MP and more immunosuppressive drugs during the first year of follow-up.

All patients received HCQ for almost the complete 5-year follow-up time (mean 57 months). The prevalence of CV risk factors and aPL positivity was similar in both groups. The mean (SD) dose of prednisone in patients in the GC5-On group was 2.96 mg/d (1.4) during the whole follow-up and 2.6 mg/d (1.2) during the 5th year (Table 1), with 75% of patients taking doses ≤ 3 mg/d during the last year of the study.

### 3.2. General Damage Accrual

As a whole, 14 patients (11%, 95%CI 6–17%) accrued damage during the 5-year follow-up time. The distribution of SDI subcategories is shown in Table 2. Lupus and GC-related damage were very unusual (1 patient each), while CV was seen in five patients (4%, 95%CI 1.2–8.6%). The proportion of patients accruing damage was evenly distributed across the 5 years of follow-up, except for year 4 with only 7% of cases. CV damage was accrued between years 3 and 5 in 4/5 patients.

### 3.3. Damage Accrual According to GC Withdrawal Groups

There was a trend for increased global damage accrual among patients in the GC5-On group: 16% vs. 7% in the GC5-Off group, *p* = 0.08 (Table 2). This trend was also seen in the unadjusted Cox regression: HR 2.5 (95%CI 0.85–7.6).

The analysis per SDI subcategory showed that the highest difference was found in CV domains, 7% vs. 1%, respectively, *p* = 0.16. The GC-related, lupus-related and other damages were evenly distributed between both groups (Table 2).

In the unadjusted Poisson regressions for global and CV-restricted final SDI scores (Table 3), no significant associations were found with being in the GC5-On group (*p* = 0.11 and *p* = 0.13, respectively).

### 3.4. Damage Accrual Analysis with PS Adjustment

In the PS-adjusted Cox analysis, the GC5-On group was not significantly associated with global damage accrual (HR 1.74, 95%CI 0.48–6.19, *p* = 0.39) or with CV damage accrual (HR 1.8, 95%CI 0.15–21, *p* = 0.62). Likewise, in the PS-adjusted Poisson models, no significant associations between the GC5-On group and the absolute and CV-restricted final SDI scores were found (*p* = 0.40 and *p* = 0.63, respectively). The PS-adjusted analyses are shown in Table 3.

## 4. Discussion

The association between GC therapy and damage is well established [1,10]. However, given that genomic effects are mainly responsible for GC toxicity, the dose is crucial for accruing damage, as is the time of exposure [11]. Doses ≤ 5 mg/d have been considered safe and are the currently recommended upper limit for maintenance therapy [6], although it is not well established whether long-term use of such low doses could also result in increased damage accrual. Given the ongoing debate over whether GC should be fully withdrawn in remission, our results support the view that low doses (<5 mg/d) may be a reasonable option for maintaining remission without contributing significantly to long-term damage accrual.

This topic has been the subject of intense research over the last few years. A French clinical trial compared the outcomes of patients in prolonged clinical remission on prednisone 5 mg/d, randomized to withdrawing or continuing on GC therapy [14]. The authors found an increased risk of flares among patients stopping GC, with no differences between both groups regarding damage accrual during the follow-up, which was however limited to one year [14]. Following this clinical trial, the systematic review by Ji et al. compiled the evidence about the effects of GC withdrawal on activity and damage in lupus patients in remission [8]. While a small increase in the flare rates among patients stopping GC was confirmed, only a borderline effect on damage prevention was seen in this group, with no observed differences in specific domains such as diabetes mellitus, osteoporotic fractures or osteonecrosis [8].

More recently, a Swedish study has suggested that any dose of GC above 0 increases the risk of damage, infections and death with a dose gradient [15]. However, this study did not consider the characteristics of each patient’s disease severity and course, nor any GC taken at higher doses during previous periods of activity, thus overestimating the effects of low-dose GC. In addition, less than half patients were treated with hydroxychloroquine, a fact that can certainly impact the degree of damage accrual [16]. In line with these results, Tselios et al. found in a propensity score-matched study of 204 patients a higher proportion of patients accruing damage among those on prolonged therapy with prednisone 5 mg/d vs. those withdrawing GC therapy (17.6% versus 6.9%), most damage being considered GC-related [17]. A major limitation of this study was that patients continuing with GC therapy had more than 5 g in excess in the previous cumulative load of prednisone, which results in an evident bias when analyzing the subsequent accrual of damage. In addition, only 65% of patients were on hydroxychloroquine [17]. Lastly, a study of the Asia-Pacific Lupus Collaboration Group, in 1850 serologically active/clinically quiescent patients, has found protection against damage accrual among those in whom GC dose was reduced by 1 mg/d from baseline doses between 5 and 7.5 mg/d (adjusted HR 0.96, 95% CI 0.94 to 0.99, vs. patients not reducing GC doses), but not in those with baseline doses ≤ 5 mg/d (HR 0.98, 95% CI 0.95 to 1.01) [18]. Therefore, the debate on the long-term safety of low-dose GC is still open.

In this study, limited to inception SLE patients fulfilling DORIS remission criteria at all the 5 yearly checkpoints after the diagnosis, we found no independent associations between low-dose GC intake during the whole follow-up and damage accrual, either global or restricted to specific domains. Although patients in the GC5-On group tended to accrue more damage, this was likely explained by the effects of a more severe disease at presentation of this subgroup, as shown in the PS-adjusted analysis. Such an effect of baseline activity on damage, irrespective of the GC dose received, has been established in previous studies [2]. Indeed, damage was accrued by a low proportion of our patients, 11% of the whole study group, 16% in the GC5-On subgroup, in comparison with more than 50% of SLICC patients within the same period of follow-up [2], highlighting the importance of achieving early and prolonged remission.

Compared to previous studies on this topic, ours is unique for a number of design characteristics. First, only patients in DORIS remission were included; therefore, all patients received, by definition, a maximum 5 mg/d of prednisone during the follow-up and lupus activity was not a relevant actor regarding damage accrual after the first year. Second, due to our therapeutic protocols already described [4,9], GC doses received during the first year, even in the GC5-On group, were substantially lower than those reported by other groups [2,17]. Third, being an inception cohort, patients were not exposed to GC prior to the study period. This is an essential point, since GC load prior to the maintenance period with low-dose prednisone, usually during more than 10 years, was not controlled for in previous studies [15,17,18]. Fourth, we extended the follow-up to 5 years, a period during which a large proportion of damage has already taken place [2]. Lastly, the use of PS analysis allowed us to minimize the potential influence of confounders for damage accrual beyond low-dose GC exposure [13], thus increasing the strength of the analysis.

Taking into account all the complexity inherent to this issue, our results align with recent studies suggesting the safety of low-dose GC in lupus patients in remission during periods of several years [14,17] and contrast with others like the Swedish cohort study suggesting that any GC dose may contribute to damage [15]. Such differences may stem from patient heterogeneity as well as from antimalarial and GC usage patterns, including past exposure to a high GC load during periods of activity. Although results regarding the increased risk of flare among patients with SLE in remission after complete GC discontinuation are conflicting [8,14,17], this possibility, with the subsequent re-introduction of prednisone at higher doses, should be taken into account before attempting withdrawal. Indeed, in terms of damage accrual, the rapid tapering of prednisone to ≤5 mg/d within a few weeks after a lupus flare is much more important than the absolute discontinuation of GC [11,19]. Therefore, the routine scalation of therapy in order to stop low-dose prednisone does not seem justified in light of recent data [18], including the results of this study. Prolonged remission with judicious use of MP, immunosuppressive drugs and antimalarials plus maintenance, if needed, with prednisone <5 mg/d, seems achievable and safe [4,9]. Slow tapering from 5 to 0 mg/d and therapy with hydroxychloroquine have been consistently identified as predictors for the successful withdrawal of GC [17,20,21,22].

This study has several limitations. The relatively small sample size and the high proportion of White patients, along with the low frequency of damage, could have resulted in a reduced power to detect associations between GC use and damage. Also, some additional damage could arise with longer follow-up times; however, as said, many patients with SLE accrue their first damage within the first 5 years of the disease course [2]. Patients in the GC5-On group were compared with patients exposed to GC for a variable period of time, but more than 70% of them stopped GC before the second year of follow-up. In general, the low incidence of damage accrual in our cohort (including the virtual absence of both lupus and GC-related damage) reinforces the efficacy and safety of a therapeutic regime based on the rapid induction of remission with MP and the generalized use of HCQ with reduced doses of prednisone since the very beginning [4,9].

## 5. Conclusions

In conclusion, our findings support the safety of maintaining low-dose prednisone (<5 mg/d) in patients with prolonged remission, as it does not appear to significantly increase the risk of damage accrual. These results suggest that, for patients in stable remission, low-dose GC therapy may be a viable strategy for long-term management without causing significant damage. The decision of discontinuing GC in this setting should be taken individually, after a careful consideration of the patient’s risk for flare, including the clinical and immunological profile and the previous history of reactivations after GC withdrawal [8,19]. The slow and progressive reduction in prednisone doses before complete removal should also be warranted [17,20,21,22]. While complete withdrawal of GC may be feasible in some cases, our results suggest that maintaining low doses can offer a protective balance between avoiding flares and minimizing long-term damage accrual.

## Figures and Tables

**Table 1:** Demographic, clinical and therapeutic variables.

Variable	Total Group (*n* = 132)	Prednisone 5-On (*n* = 56)	Prednisone 5-Off (*n* = 76)	*p*
Centre				
Cruces	109 (83%)	51 (91%)	58 (76%)	0.03
Bordeaux	23 (17%)	5 (9%)	18 (24%)	
Female	110 (83%)	43 (77%)	67 (88%)	0.08
White	126 (95%)	54 (96%)	72 (95%)	0.64
CVRF	32 (24%)	14 (25%)	18 (24%)	0.86
aPL positivity	46 (35%)	22 (39%)	24 (31%)	0.36
Baseline SLEDAI, mean (SD)	5.3 (4.1)	6.3 (5.1)	4.6 (2.9)	0.01
Categorical baseline SLEDAI				
<6	80 (60%)	29 (52%)	51 (67%)	0.004
6–12	45 (34%)	20 (36%)	25 (33%)	
>12	7 (5%)	7 (12%)	0 (0%)	
Prednisone year 1, maximum dose (mg/d), mean (SD)	9.5 (12)	12.4 (11)	7.4 (13)	0.02
Prednisone year 1, daily dose (mg/d), mean (SD)	3.06 (3.7)	4.2 (3.4)	2.18 (3.6)	0.001
Prednisone years 1–5, daily dose (mg/d), mean (SD)	1.67 (1.7)	2.96 (1.4)	0.72 (1.16)	<0.001
Prednisone year 5, daily dose (mg/d), mean (SD)	1.12 (1.5)	2.65 (1.3)	0	<0.001
MP year 1	39 (30%)	26 (46%)	13 (17%)	<0.001
Total time on HCQ (months), mean (SD)	57 (9)	57 (9)	57 (9)	0.88
Oral IS year 1	27 (20%)	31 (55%)	6 (8%)	<0.001
CYC year 1	6 (5%)	6 (11%)	0 (0%)	0.003

SD: standard deviation. CVRF: cardiovascular risk factors. aPL: antiphospholipid antibodies. CNS: central nervous system. APS: antiphospholipid syndrome. SLEDAI: systemic lupus erythematosus disease activity index. HCQ: hydroxychloroquine. MP: methyl-prednisolone pulses. IS: immunosuppressives. CYC: cyclophosphamide.

**Table 2:** Distribution of damage.

Type of Damage	Total Group (*n* = 132)	Prednisone 5-On (*n* = 56)	Prednisone 5-Off (*n* = 76)	*p*
Global damage	14 (11%, 95%CI 6–17%)	9 (16%, 95%CI 7.6–28%)	5 (7%, 95%CI 2.1–14%)	0.08
CV damage	5 (4%, 95%CI 1.2–8.6%)	4 (7%, 95%CI 2–17%)	1 (1.3%, 95%CI 0.3–7%)	0.16
GC damage	1 (0.8%, 95%CI 0.1–4%)	0 (0%)	1 (1.3%, 95%CI 0.3–7%)	1
Lupus damage	1 (0.8%, 95%CI 0.02–4%)	1 (1.8%, 95%CI 0.4–9%)	0 (0%)	0.4
Other damage	7 (5.3%, 95%CI 2–11%)	4 (7%, 95%CI 2–17%)	3 (4%, 95%CI 0.8–11%)	0.4

CV: cardiovascular. GC: glucocorticoid.

**Table 3:** Cox and Poisson regression models (independent variable GC5-On group).

**Cox Regression**				
**Type of Damage**	**Without PS Adjustment**	***p***	With PS * Adjustment	***p***
Global damage	HR 2.5 (95%CI 0.85–7.6)	0.09	HR 1.74 (95%CI 0.48–6.19)	0.39
CV damage	HR 5.4 (95%CI 0.61–48.9)	0.13	HR 1.8 (95%CI 0.15–21)	0.62
**Poisson Regression**				
**Type of Damage**	**Without PS Adjustment**	***p***	With PS * Adjustment	***p***
Global damage	Coefficient 0.89	0.11	Coefficient 0.54	0.40
CV damage	Coefficient 1.7	0.13	Coefficient 0.58	0.63

GC5-On group: on low-dose glucocorticoid therapy during the 5-year follow-up. PS: propensity score. CV: cardiovascular. * The C-index of the propensity score model was 0.79.

## Data Availability

The data that support the findings of this study are available on request from the corresponding author. The data are not publicly available due to privacy or ethical restrictions.
